# A Personalized Computer-Aided Diagnosis System for Mild Cognitive Impairment (MCI) Using Structural MRI (sMRI)

**DOI:** 10.3390/s21165416

**Published:** 2021-08-11

**Authors:** Fatma El-Zahraa A. El-Gamal, Mohammed Elmogy, Ali Mahmoud, Ahmed Shalaby, Andrew E. Switala, Mohammed Ghazal, Hassan Soliman, Ahmed Atwan, Norah Saleh Alghamdi, Gregory Neal Barnes, Ayman El-Baz

**Affiliations:** 1 Bioengineering Department, University of Louisville, Louisville, KY 40292, USA; fatma_zahraa@mans.edu.eg (F.E.-Z.A.E.-G.); ahmahm01@louisville.edu (A.M.); ahmed.shalaby@louisville.edu (A.S.); andy.switala@louisville.edu (A.E.S.); aselba01@louisville.edu (A.E.-B.); 2 Information Technology Department, Faculty of Computers and Information, Mansoura University, Mansoura 35516, Egypt; melmogy@mans.edu.eg (M.E.); hsoliman@mans.edu.eg (H.S.); ahmed.atwan@nbu.edu.sa (A.A.); 3 Department of Electrical and Computer Engineering, Abu Dhabi University, Abu Dhabi 59911, United Arab Emirates; mohammed.ghazal@adu.ac.ae; 4 College of Computer and Information Science, Princess Nourah Bint Abdulrahman University, Riyadh 11564, Saudi Arabia; 5 Department of Neurology, University of Louisville, Louisville, KY 40292, USA; gregory.barnes@louisville.edu

**Keywords:** Alzheimer’s disease, personalized diagnosis, mild cognitive impairment, computer-aided diagnosis, sMRI

## Abstract

Alzheimer’s disease (AD) is a neurodegenerative disorder that targets the central nervous system (CNS). Statistics show that more than five million people in America face this disease. Several factors hinder diagnosis at an early stage, in particular, the divergence of 10–15 years between the onset of the underlying neuropathological changes and patients becoming symptomatic. This study surveyed patients with mild cognitive impairment (MCI), who were at risk of conversion to AD, with a local/regional-based computer-aided diagnosis system. The described system allowed for visualization of the disorder’s effect on cerebral cortical regions individually. The CAD system consists of four steps: (1) preprocess the scans and extract the cortex, (2) reconstruct the cortex and extract shape-based features, (3) fuse the extracted features, and (4) perform two levels of diagnosis: cortical region-based followed by global. The experimental results showed an encouraging performance of the proposed system when compared with related work, with a maximum accuracy of 86.30%, specificity 88.33%, and sensitivity 84.88%. Behavioral and cognitive correlations identified brain regions involved in language, executive function/cognition, and memory in MCI subjects, which regions are also involved in the neuropathology of AD.

## 1. Introduction

Alzheimer’s disease (AD) is considered the best-known neurodegenerative conditions targeting the central nervous system (CNS). Elderly people make up the preponderance of the sufferers of AD. However, younger people may be affected by early-onset AD [1]. Statistically speaking, disease risk increases with age among the elderly population, with 42% of those diagnosed with AD being 85 years or older, while only 6% of diagnosed cases are between 70 and 74 years old [2].

The characteristics of AD can be broadly grouped into clinical and anatomical features [3]. Features in either category vary from one patient to another. Clinically, AD patients show progressive deficits in cognition and memory in addition to disturbances in thought, perception, and behavior. Pathologically, patients incur a neuronal loss, granulovacuolar degeneration, and the formation of the two definitive diagnostic markers of AD: neurofibrillary tangles and neuritic plaques [4]. Up-regulated expression of the amyloid-β precursor protein (APP) is followed by a cascade of processing involving BACE1, PSEN1, PSEN2, and APH1, resulting in production of amyloid-β peptide, including its pathogenic species Aβ42. The Aβ42 conformations fuse into oligomers containing up to 100 units of Aβ42, and form neurotoxic protofibrils. Aβ42 oligomers itself leads to synaptic loss, neurotoxicity, and neuronal death. Aβ42 oligomers, under the influence of ApoE4, can undergo aggregation and formation of Aβ seniles plaques in affected brain regions [5].

As a neurodegenerative condition, AD is progressive. The severity of affliction is typically divided into three phases, beginning with a mild phase, then proceeding to moderate phase, and ending with severe phase [6]. The emergence of the disease’s pathological features 10–15 years before being clinically discovered hinders the early diagnosis of the disease. Furthermore, the subject-dependent influence of AD between its sufferers adds another obstacle to diagnosing the disease in its early stage [4].

Various tests of a patient’s mental and physical state can assist in AD diagnosis, including urinalysis, blood panels, and neurological, neuropsychological, psychiatric examinations. The patient’s medical history, as well as brain imaging in various modalities, can also inform the diagnosis [1]. Regarding brain imaging, these technologies play a notable role in identifying the disease, specifically speaking in the pre-clinical and MCI phases [7]. Further information about the impact of brain imaging in this research area can be found in the study presented by Johnson et al. [8]. Additionally, a scientific work presented by Jack et al. [9] aimed to illustrate the function of each of the brain biomarkers along the cascade of AD. Relying on the study findings, for the earliest signs of the disease, positron emission tomography (PET) amyloid imaging, as well as cerebrospinal fluid (CSF) levels of amyloid beta (A$\beta_{42}$), reveal evidence of the underlying Aβ pathology. CSF levels of tau protein, structural magnetic resonance imaging (sMRI), 2-[18F] fluoro-2-deoxy-d-glucose (FDG-PET), and the cognitive and clinical symptoms can help follow patients as pathology accumulates with disease progression. sMRI discloses the structural abnormalities while FDG-PET or CSF-tau reveal neuronal injury and dysfunction.

Previous scientific research has attempted, through several methodologies, to different groups defined by cognitive status (normal control (NC), MCI, or AD) using neuroimaging data. For instance, a computer-assisted diagnostic (CAD) system was presented in [10] to diagnose AD at its earliest phase using independent component analysis (ICA) as well as support vector machines (SVM) for the feature extraction and the classification purposes, respectively. Additionally, a CAD system using Gaussian discriminant analysis was presented in [11] to screen the disease’s phases where the features of the entorhinal cortex showed significant discriminatory power between both the normal group (NC) and abnormal group (MCI + AD). Additionally, the study could achieve an improvement regarding the classification performance through defining two separate spaces of the decision, for both hemispheres of the brain (left and right hemispheres), following by combining their obtained result. Beheshti et al. [12] used feature ranking in addition to genetic algorithms (GA) to propose a CAD system that addressed differentiating between NC, stable MCI (sMCI), progressive MCI (pMCI), as well as AD groups. The pMCI group comprises subjects who progressed clinically to the overt AD where their neuropsychological tests have a poorer performance than the NC group.

On the other hand, the sMCI, who either remains in the stable stage or may improve, shows no or marginal neuropsychological changes [13,14]. Zhang et al. [15] addressed the three-way classification problem between the NC, MCI, and AD groups. In this system, the principle analysis is used for feature detection, while the kernel support vector machine decision tree (kSVM-DT) was used for the classification purpose. Then, Zhang et al. [16] used the idea behind the eigenbrains along with the machine learning for building their CAD system. Therefore, Welch’s t-test was used to find significant eigenbrain while the prediction task was accomplished using SVM with the implementation of different kernels. Tong et al. [17] exploited the multiple instance learning (MIL) method to present a system aimed to diagnose both AD and MCI phases. In this system, the extracted features were in the form of local intensity patches. The MIL method was applied to address the case when some patches may not characterize the morphological association with AD because of the variable influence of the disease on these patches. Finally, Westman et al. [18] used orthogonal partial least squares to latent structures (OPLS) analysis to discriminate between the groups of AD through combining local and global volumetric measures obtained from MRI scans.

Despite the achievements mentioned above, there are several notes regarding these achievements that led to making the door still open in front of this research topic, and specifically speaking this AD-related research point (i.e., differentiating between NC and MCI groups). First, the previously mentioned studies addressed either a diagnosis of whole-brain findings consistent with impairment or else considered local, brain region-specific diagnosis while excluding the MCI group. Despite the importance of those researchers’ findings in the diagnosis task, targeting the brain-based regional diagnosis might add more advantages due to the disease’s subject-dependent influence that could impede the early diagnosis. Furthermore, the local/regional diagnosis can aid in revealing the disease-related ambiguity. Secondly, in general, the diagnosis performance when using sMRI in the AD early stage is fair and still needs more improvements. Due to the literature, the sMRI scans can be used to follow patients as pathology accumulates with disease progression. In contrast, at the early stages, the scan might look normal [9,19]. The aim of this paper is primarily to introduce a system for the local/regional diagnosis, using sMRI technology, for serving the goal of personalized diagnosis of MCI. Therefore, the proposed system studies the impact of MCI locally (i.e., in the term of the local brain regions), specifically speaking its impact on the brain cortical regions. Targeting the cortical regions is due to the essential role of the medical imaging-based measurement of the cerebral cortex’s shape, composition, as well as function in the diagnosis of the neurodegenerative conditions and explicitly speaking in diagnosing AD [20,21,22]. To support the performed cortical regions diagnosis, further analysis of the obtained results has been performed to confirm the fitness of the results with the neurocircuits defined by the National Institute of Mental Health Research Domain Criteria (RDoC). In addition, the paper offers a global diagnosis where the results are promising, as evaluated, in addressing the challenging task of differentiating between the NC and MCI groups primarily through brain structuring features at the early stage of the disease. This paper is organized as follows. Section 2 explains the used material as well as the applied methods. Section 3 presents the evaluation results of the proposed CAD system. Section 4 discusses the obtained findings. In the end, a conclusion of the proposed study is shown in Section 5.

## 2. Materials and Methods

### 2.1. Materials

Data from the Alzheimer’s Disease Neuroimaging Initiative (ADNI) database (adni.loni.usc.edu, (Last accessed on 1 July 2021)) was used to build the proposed system. ADNI is considered to be a standard database, which was established in 2003 as a public-private partnership under the lead of Michael W. Weiner, MD as a Principal Investigator. The aim behind the ADNI was to evaluate the role of combining serial MRI, PET, or other markers, along with the clinical and neuropsychological assessments, in measuring the evolution of MCI as well as AD. All the data on ADNI are provided for both the informational as well as the review purposes where according to ADNI, the IRB in approved for research use only. In the proposed work, we used 146 baseline sMRI scans of 60 normal plus 86 mildly cognitively impaired subjects, classified in ADNI as being either sMCI or pMCI. Table 1 shows the demographic distribution of the used dataset. As reported by ADNI, the NC participants represent the control subjects who do not show any depression, MCI, or dementia signs. On the other hand, the MCI subjects are the subjects with subjective memory concern that is reported by an informant, a clinician, or oneself. Despite this reported concern, the daily living activities of the MCI participants are basically preserved. The subjects neither show any significant impairment levels in other cognitive domains nor show dementia signs [23]. Please note here that in our paper, we did not focus on differentiating between the sMCI and the pMCI groups. This is due to our ultimate goal of presenting a personalized CAD system of either belonging to the NC or the MCI group without addressing whether the subject will proceed to AD, as in the pMCI group, or will remain stable, as in the sMCI group.

### 2.2. Methods

**Figure 1:**
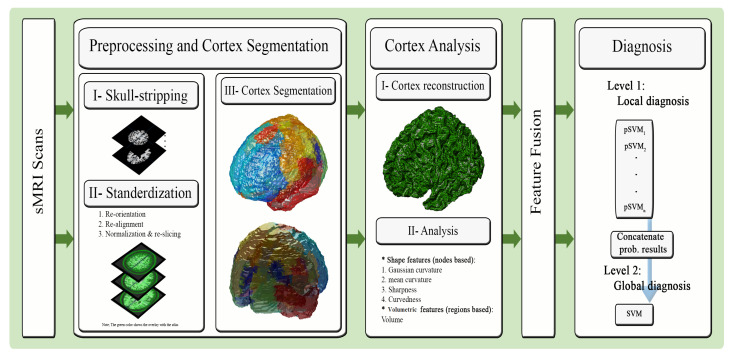
The proposed cortical region-based diagnostic system of cognitive impairment using sMRI.

This paper aims to present a cortical region-based CAD system to perform the personalized diagnosis of MCI through the framework illustrated in Figure 1. The system begins with preprocessing the scans as well as segmenting the cerebral cortex and parcellating by hemisphere. Second, a triangular mesh reconstruction of the cortical surface is performed using the marching cubes (MC) algorithm. This is followed by the extraction of shape-based features at each node of the cortical mesh. The cortical region-based features are then defined through applying the Automated Anatomical Labeling (AAL) atlas to the reconstructed cortex. Third, a fusion of the obtained features is performed using canonical correlation analysis (CCA) to produce more representative features. Fourth, a two-stage diagnostic classifier is constructed, producing cortical region-specific diagnoses that are combined into a final diagnosis, of the subject’s cognitive status.

#### 2.2.1. Preprocessing and Brain Cortex Segmentation

**Figure 2:**
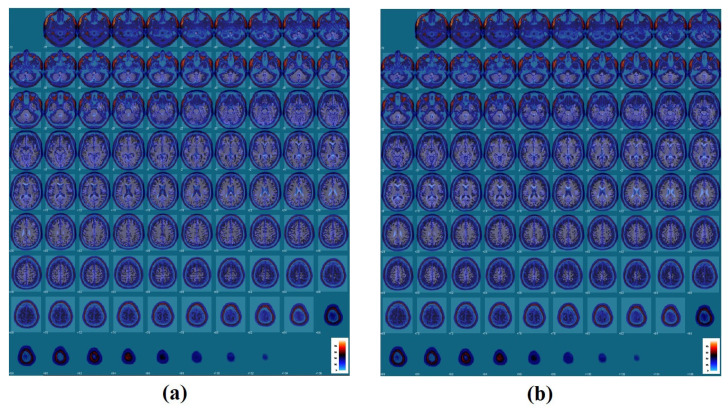
Example of preprocessed and overlaid subjects’ scans with the AAL atlas template from each studied group where (**a**) is for a normal subject, while (**b**) is for a mildly cognitive impairment subject).

This step serves the cortical regions-based diagnosis goal through standardizing them to the parcellation atlas space. Using the SPM toolbox, images are resampled and re-oriented (if necessary), skull-stripped, aligned, and spatially normalized. Skull stripping in this case had already been performed, so we convolved the sMRI scans with their corresponding brain masks that in turn are provided as part of the ADNI dataset. Then, the orientation of the atlas template’s space, MNI space, had been matched with the scans through re-aligning re-orientating, spatial normalize as well as re-slicing the scans. The data were re-sliced and aligned with the MNI-152 standard template. One scan, selected as a reference, was rotated and shifted to align as near as possible to the template, with the line between the anterior and posterior commissures (AC-PC line) of the template and reference aligning exactly. The rest of the scans in the dataset were registered to the chosen reference with a rigid body transformation calculated to optimize the mutual information criterion. The particular choice of reference image is not significant, since all MRI in the ADNI database have roughly the same spatial orientation. Subsequently, the algorithm of Ashburner and Friston [24] was used to register each pre-aligned image precisely with the MNI-152 template using a combination of affine and nonlinear deformations. Figure 2 shows examples of preprocessed scans overlaid on the atlas template [25]. Following this step, segmentation of the cerebral cortex was performed using the xjview MATLAB toolbox.

#### 2.2.2. Brain Cortex Reconstruction and Analysis

**Figure 3:**
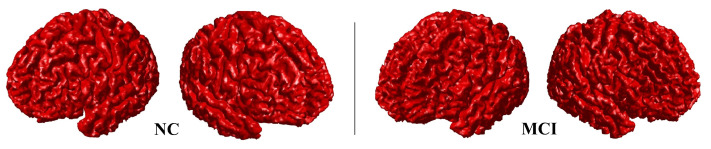
Examples of the marching cubes reconstruction output for normal and mildly cognitively impaired subjects. As shown, although it is not that obvious since it is still the early stage of the disorder, the brain atrophy starts to take place in the MCI case, where this atrophy defines the beginning of losing the neurons and the connections that exist between them.

The shape descriptors to be used later by the algorithm depend upon the accurate representation of the cortical surface. Therefore, the MC algorithm is initially used for cortex reconstruction since it is best-known isosurface extraction method and produces high-resolution results [26,27]. Then, having obtained the triangulated mesh representation of the cortical surface, several shape features are calculated at each node individually through Equations (1)–(4) after calculating the principal curvature directions and values. Algorithm 1 summarizes the steps of the MC algorithm as well as the calculation of the principal curvature directions and values while Figure 3 illustrates results of cortical surface reconstruction for both NC and MCI subjects.
**Algorithm 1** The MC algorithm and the calculation of the principal curvature directions and values.**Input:** The dataset of the scalar volumetric**Output:** The directions and values of the principle curvature**Steps:**Use the volume lattice for defining the cubes ($C_{l}$) in which the corner vertices are defined through the points ($P(x_{i},y_{j},s_{k})$) of the lattice for the column $x_{i}\left(\forall_{i}\right)$, $y_{i}\left(\forall_{j}\right)$ and the slice $S_{k}\left(\forall_{n}\right)$ where n represent the number of the volume slices.Construct, in a sequential form of cube-by-cube manner throughout the rows of the dataset, a fecetized isosurface. In this procedure and when the value of the $V_{i}\;\geq$ isovalue (α), mark $V_{i}$ and keep the remaining ones as unmarked. Consequently, the “active” edges are defined as an edge ($E_{j}$) ended with a marked vertex ($V_{j}m$) and an unmarked vertex ($V_{j}u$). Note: the value of α was calculated through applying the histogram to the labeled volume, remove the large first max value, and obtain the value of a middle bar of non-small values as the α value.Use a look-up table to factorize the interacted isosurface of the intersection topologies in which the linear interpolation is applied for the location estimation of the intersection between the isosurface-edge through:
$$I\left(x,y,s\right)=V_{m(x,y,s)}+\rho \left(V_{u(x,y,s)}-V_{m(x,y,s)}\right)$$
where: $\rho =\frac{\alpha -L_{m}}{L_{u}-L_{m}}$, $L_{m}$ and $L_{u}$ are the scalars values $V_{m}$ as well as $V_{u}$, respectively.Through the face and vertex lists of the resulting triangulated mesh and to calculate the principal curvature directions and values, describe the input by XY rather than XYZ through rotating the input so the current vertex’s normal becomes [−1 0 0].Fit a patch of the least-squares quadratic to the local neighborhood of a vertex “$f\left(x,y\right)=ax^{2}+by^{2}+cxy+dx+ey+f$”.Use the hessian-based eigenvectors and eigenvalues to calculate the principal curvature.

Please note that the sharpness and curvedness features were used as in [28]. Next, labeling of each of the mesh nodes to its corresponding cortical regions is performed using the AAL atlas, which defines a total of 76 cortical regions. It is important to note here that alternative brain parcellation schemes could be used, as in [15,29,30]. In the proposed system, the AAL atlas was chosen because of its relatively fine granularity. Here, to make sure of the matching between the labels and the surface, the preprocessing steps of the proposed framework were first applied to standardize the scans to the geometry of the atlas template’s space, MNI space. Then, converts MNI coordinate to a description of brain structure in AAL atlas using a standard list of the MNI space of the parcellation atlas to label the required brain cortical regions.

(1) $$C_{\mathrm{Gaussian}}=\lambda_{1}\lambda_{2}$$

(2) $$C_{\mathrm{mean}}=\frac{1}{2}\left(\lambda_{1}+\lambda_{2}\right)$$

(3) $$\mathrm{Sharpness}={\left(\lambda_{1}-\lambda_{2}\right)}^{2}$$

(4) $$\mathrm{Curvedness}=\sqrt{(\lambda_{1}^{2}+\lambda_{2}^{2})/2}$$

where $\lambda_{1}$ and $\lambda_{2}$ denote the principal curvatures. Quantities are estimated at the locus of each node of the triangulated surface.

Although grey matter volume has a significant impact in the AD research area, where it is considered to be the most popular cross-sectional quantitative metric [31], the demographic variability between the subjects can bias results. For this reason, the volume is used here in conjunction with the previously obtained features to increase the precision of the results while avoiding this biasing possibility. To calculate the volume: (1) apply the AAL atlas to the to the preprocessed scans to define the cortical regions of the brain, (2) the MC algorithm is applied to reconstruct each region separately, (3) calculate the volume for each of the reconstructed regions separately. By the end of this step, there are a total of five features calculated for each of the 76 brain cortical regions, and they are now ready for the next step of fusion.

#### 2.2.3. Shape Feature Fusion

This step aims to fuse the previously extracted features to produce more informative discriminative features between the tested groups. For this purpose, the CCA-based technique of feature fusion is used due to its role in finding the associations between two sets of variables [32]. Obtaining the linear combinations helps in discovering this association that consequently enlarge the correlation between the two variable sets in the way that presented in Algorithm 2. Here and due to the number of studied features, five features, the CCA technique is implemented sequentially working with two features at a time until ending up with the final fusion-based feature vector for each labeled region. Note, due to the different scales of the extracted features, before fuse the features using the CCA technique, each of the features are normalized to be between 0 and 1 using Equation (5).

(5) $$normFeat=\left(oldFeat-oldFeat_{min}\right)/\left(oldFeat_{max}-oldFeat_{min}\right)$$

**Algorithm 2** The algorithm for feature fusion based on CCA technique.
**Input:** Two matrices of the features, $X\in R^{p\times n}$ and $Y\in R^{q\times n}$, of the extracted (p+q) features for the *n* samples.
**Output:** The fused features in the form of matrix.
**Steps:**Compute the covariance matrix, *S*, for the two matrices *X* and *Y* using:
$$S=(\begin{array}{cc} cov(x) & cov(x,y)\\ cov(y,x) & cov(y) \end{array})=(\begin{array}{cc} S_{xx} & S_{xy}\\ S_{yx} & S_{yy} \end{array})$$
where the $S_{xx}\in R^{p\times p}$ and the $S_{yy}\in R^{q\times q}$ are within-sets matrices of the covariance of the X as well as the Y, respectively. The $S_{xy}\in R^{p\times q}$ is the matrix of the between-set covariance while $S_{yx}=S_{xy}^{T}$Determine both of the linear combinations $X^{*}$ and $Y^{*}$ through using CCA to be able to enlarge the correlations among the matrices *X* and *Y* through:
$$corr\left(X^{*},Y^{*}\right)=\frac{cov(X^{*},Y^{*})}{var\left(X^{*}\right).var\left(Y^{*}\right)}$$where $W_{x}$ and $W_{y}$ represent the matrices of the transformation. $cov\left(X^{*},Y^{*}\right)=W_{x}^{T}S_{xy}W_{y}$, $var\left(X^{*}\right)=W_{x}^{T}S_{xx}W_{x}$, and $var\left(Y^{*}\right)=W_{y}^{T}S_{yy}W_{y}$. The usage of Lagrange multipliers is to attain the maximization goal by maximizing $cov(X^{*},Y^{*})$ with a constrain of $var\left(X^{*}\right)=var\left(Y^{*}\right)=1$.Determine $W_{x}$ and $W_{y}$ by:
(a)Solve the equations of the eigenvalue:
$$S_{xx}^{-1}S_{xy}S_{yy}^{-1}S_{yx}\hat{W_{x}}=\Delta^{2}\hat{W_{x}}$$
where $\hat{W_{x}}$ and $\hat{W_{y}}$ are the eigenvectors while $\Delta^{2}$ is the eigenvalues that corresponds to either the diagonal matrix or the canonical correlations square.(b)Determine *d* that represent the overall non-zero eigenvalues in every aforementioned equation, by $d=rank(S_{xy}\left(n,p,q\right))$.(c)Perform a decreasing order-based sorting operation of the previous step results $\delta_{1}\geq \delta_{2}\geq \ldots \geq \delta_{d}$.(d)Let the sorted eigenvectors be indicated by $W_{x}$ and $W_{y}$ where they consequently represent the non-zero eigenvalues in which $X^{*}$ and $Y^{*}\in R^{dn}$ represent the canonical variates.Calculate the sample covariance matrix of the transformed data, $S^{*}$, using:
$$S^{*}=\left[\begin{array}{cccc} 1 & 0 & \cdots & 0\\ 0 & 1 & \cdots & 0\\ \vdots & & \ddots & \vdots \\ 0 & 0 & \cdots & 1\\ \delta_{1} & 0 & \cdots & 0\\ 0 & \delta_{2} & \cdots & 0\\ \vdots & & \ddots & \vdots \\ 0 & 0 & \cdots & \delta_{d} \end{array}|\begin{array}{cccc} \delta_{1} & 0 & \cdots & 0\\ 0 & \delta_{2} & \cdots & 0\\ \vdots & & \ddots & \vdots \\ 0 & 0 & \cdots & \delta_{d}\\ 1 & 0 & \cdots & 0\\ 0 & 1 & \cdots & 0\\ \vdots & & \ddots & \vdots \\ 0 & 0 & \cdots & 1 \end{array}\right]$$
Concatenate the features-based transformed vectors to obtain the feature fusion vector through:
$$Z=(\begin{array}{c} X^{*}\\ Y^{*} \end{array})=(\begin{array}{c} W_{x}^{T}X\\ W_{y}^{T}Y \end{array})={\left(\begin{array}{cc} W_{x} & 0\\ 0 & W_{y} \end{array}\right)}^{T}(\begin{array}{c} X\\ Y \end{array})$$


#### 2.2.4. Diagnosis

The last step of the proposed system is to use the fused features to train the two diagnostic layers: regional and global. For this purpose, a probabilistic SVM (pSVM) support vector machine (pSVM) is used in the first diagnosis layer, where for each anatomical region a separate pSVM is trained to produce a probabilistic measure of association of that particular region’s features with MCI. For this purpose, the fusion feature vector produced by the CCA technique was used as an input to the pSVM to produce the final probabilistic regional diagnosis result. Then, a standard SVM is used, in the second layer, where the probabilistic outputs of the first layer are input to it, and the output is the global diagnosis of NC or MCI.

## 3. Results

The system was trained and tested using the 146 baseline scans, previously mentioned, downloaded from ADNI. For the evaluation process, three types of experiments are performed: (1) evaluating the performance of different SVM kernels, (2) comparing the system’s performance results with several some state-of-the-art methods, and (3) validating it with related work.

**Figure 4:**
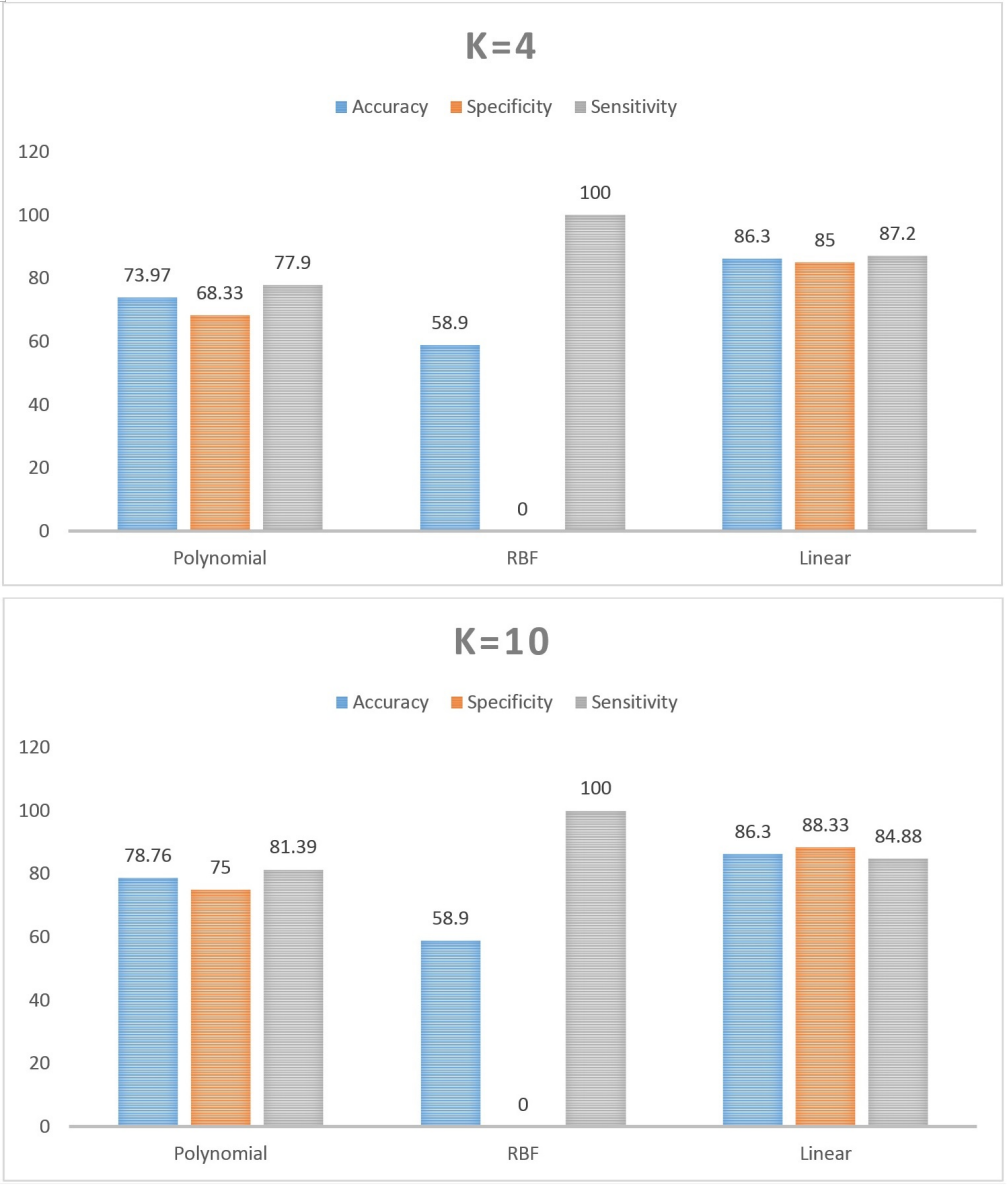
The results of the k-fold validation method in (%) for different SVM-based kernels.

**Figure 5:**
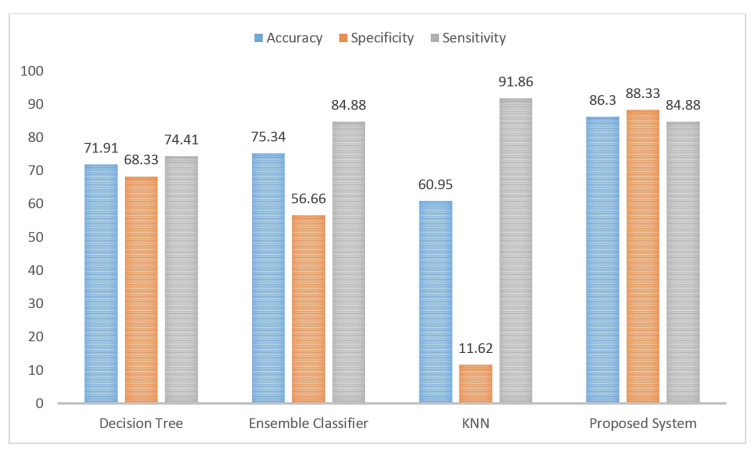
The comparison evaluation of our linear-based CAD system with some state-of-the-art classifiers with k-fold = 10.

For testing classifier performance, k-fold cross-validation was applied to compare both the results of the SVM-related kernels, as shown in Figure 4, and our obtained results against some state-of-the-art methods, as shown in Figure 5. Regarding the k-fold cross-validation method, K = 4 and K = 10 were used to verify that the proposed system did not overfit while K = 10 was also used to evaluate the proposed linear-based CAD system with some state-of-the-art classifiers. As illustrated in Figure 4, the linear kernel could, in general, exceeds the overall performance of the other kernels (i.e., polynomial, and radial basis function (RBF) kernels) with the K = 4, and K = 10. For K = 4, the superior results of the linear kernel were around 86.3%, 85%, and 87.2% for the accuracy, specificity, and sensitivity, respectively. For K = 10, these superior results were around 86.3%, 88.33%, and 84.88% of accuracy, specificity, and sensitivity, respectively. Comparing the obtained results, at K = 10, with some other state-of-the-art classifiers (i.e., decision tree, ensemble classifier, and K nearest neighbors (KNN)), Figure 5, also showed that the linear-SVM generally could achieve better results.

**Figure 6:**
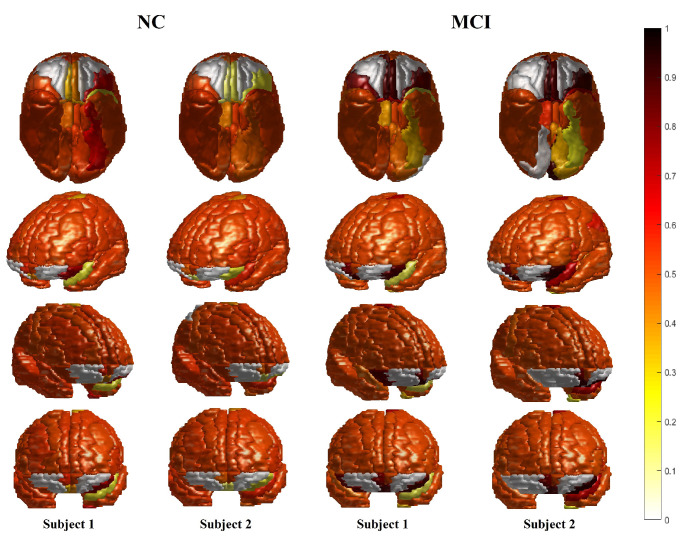
Different examples that show the cortical regions diagnosis for two different normal, and two different mildly cognitive subjects. Note: (1) the color-bar-based gradient colors represent the disease’s severity in every studied region separately. (2) The blue arrows show examples of the cortical regions that show significant difference in the probabilistic diagnosis results between the NC and MCI subjects.

Along with these quantitative performance results, an additional investigation has been performed to confirm the fitness of the obtained subjects’ cortical regions-based diagnosis results with the neurocircuits defined by the National Institute of Mental Health RDoC. Therefore, Table 2 displays the modest correlations between the behavioral and cognitive data from ADNI and critical brain regions involved in memory and language. Finally, an illustration of different cortical region-based diagnoses is presented in Figure 6 where the disease’s severity in each cortical region is represented in color.

## 4. Discussion

Patients with mild cognitive impairment present with markedly reduced cognitive abilities when compared with unaffected people of the same age, and taking a level of education into account, but without meeting the criteria for dementia. One or more domains of cognition can be influenced by this impairment: memory, executive function, language, skills of the visuospatial domain, or attention. Regardless of the aforementioned impairments, the patients still can accomplish their daily tasks, such as occupational or social functions without confusion [33]. Therefore, MCI is considered to be an intermediate condition between typically seen age-related changes in cognition and dementia [33,34]. Although it is not guaranteed that all MCI cases proceed to AD, suffering from MCI increases the risk factor of ending up with AD [34,35].

To date, sMRI is one of the most developed modalities used for differential pathological diagnosis purposes due to its ability to detect the location and severity of atrophy through showing the detailed description of the soft tissues of the body [36,37]. sMRI can discriminate between tissue types through capturing proton density or magnetization properties (using spin-spin (T2) or spin-lattice (T1) relaxation times). Actually, T1-weighted, as well as T2-weighted images, are used for qualitative assessment that is designed to both differentiate between the tissues with a different relaxation time of T1/T2, and to evaluate the macroscopic lesions as well as tissues changes such as in sulci, cysts and ventricles [38].

Regarding AD, sMRI can, in general, reveals atrophy of the cerebral cortex during the progression of AD. Furthermore, the regions thought to distinguish AD from MCI and normal controls include MRI parameters of the putative earlier involved MCI regions (hippocampus, entorhinal cortex, supramarginal gyrus) vs. earlier involved AD regions (rate of hippocampal atrophy, cingulate cortex, and parietal cortex) [39]. Additionally, the analysis of sMRI helps in uncovering the relationship between both the elevated risks for MCI converting to AD and atrophy where this, in turn, assists in anticipating the future cognitive-based decline in the healthy adults. Additionally, the volumetric-based analysis using sMRI can aid in detecting crucial changes in the brain regions’ size that in turn, effectively assist in the diagnosis procedure [40].

According to the literature, the shape, composition, and function of the cerebral cortex as measured by imaging modalities has a crucial role in diagnosing the neurodegenerative conditions, especially in AD [20,21,22]. Depending on imaging variability and due to the variability of AD effect among its sufferers, the ultimate goal of this paper is to introduce a cortical region-based diagnosis of MCI. Additionally, the paper aims to improve the overall performance of the discrimination between the NC and MCI Group, which is known to be a difficult task, as seen in the related literature.

We introduced a cortical region-based diagnostic system that serves the subject-dependent (i.e., personalized) diagnosis of MCI. Additionally, we target improving the diagnostic performance with respect to the related work. To achieve our goals, and because of the nature of the disease at this early stage, when underlying anatomical changes are subtle, it was necessary to choose high-resolution methods to accomplish this task. Therefore, in the proposed system, the MC algorithm was selected due to its role, as mentioned above in obtaining high-resolution extraction of isosurface results. Then, the shape-based features were addressed to serve the discrimination goal due to the nature of the disease’s influence in the brain that could be detected through the sMRI scans. After obtaining these features and to present a more informative feature vector to the diagnosis step, as well as to overcome the biased results that can be obtained using the volume feature, a feature reduction/fusion process was applied. Finally, and based on its powerful role in addressing this type of problems as well as to serve the personalized diagnosis role, standard SVM and its variant, pSVM, was applied to provide two layers, regional followed by global diagnosis.

As previously mentioned, the system’s performance has been evaluated from three different perspectives, which are evaluating the performance of different SVM kernels, comparing the obtained performance results with several state-of-the-art methods, and validating the system’s performance with related work. Starting with the first evaluation, Figure 4 shows a comparison of different SVM-related kernels’ performance (i.e., polynomial, linear, and RBF) using k-fold cross-validation method, with K = 4, and K = 10 to exclude the possibility of overfitting, As shown in the figure, the linear kernel achieved superior results while the RBF kernel performed most poorly. The results of the linear-SVM reflect the power of the extracted features in providing linear separation between the tested groups. On the other hand, the low results of the nonlinear kernels can be justified as the result of the small dataset size that led to lower performance results of RBF-based SVM compared with the polynomial-based one. Additionally, the power of the extracted features that caused the superior results of the linear-SVM showed, as shown in the results that the RBF kernel failed to find a proper separating decision boundary between the studied groups.

Then, again through using the k-fold cross-validation method and specifically speaking K = 10, we compared the performance of the linear-SVM with some well-known methods (i.e., decision tree, ensemble classifier, and KNN), as presented in Figure 5. Broadly speaking, the linear-SVM showed better performance against the other methods. This indicates the proposed work’s ability to deal with this research issue. In general, this better performance can be justified by several reasons. First, the discriminative power of the features that results in better classification performance ability of the linear-SVM to separate between the groups with linear hyperplanes. Second, the performance power of SVM, in general, to deal with high-dimensional space’s dataset while this is not the case with other methods. Finally, the efficiency of SVM to deal with a small size of the datasets while other methods can suffer from under-performance results and/or overfitting.

Additionally, validating our system’s performance against the literature showed the promise of the proposed work. For instance, in [15] a classification system was built, using the principal component analysis (PCA) kSVM-DT, and could reach a maximum accuracy result of 85%, specificity result of 80%, and sensitivity results of 87%. In [18], the OPLS analysis was used that led to a specificity result of 73% as well as a sensitivity result of 66%. Finally, in [41], an ICA/SVM system was proposed for the classification and could achieve accuracy, specificity, and sensitivity of 70.19%, 67.49%, and 72.89%, respectively. It is noteworthy that the results of the systems above have been obtained from those studies regardless of using different dataset as well as a different number of scans. The idea here is to validate our work against prior work focusing on the same research area.

The modest correlations between ADNI behavioral and cognitive data and brain regions (Table 2) critical to AD, involving memory and language, adds further validation to our approach. (Additional details about the ADNI categories can be found in [42,43,44,45,46,47].) Furthermore, a survey of statistically significant correlations between ADNI behavioral and cognitive data and brain regions suggest that regions linked to specific deficits in language (15 regions), executive function and cognition (10 regions), adaptive behavior (5 regions), and memory (3 regions) may point to early neuropathology in classic AD-involved regions in MCI subjects. Finally, Figure 6 illustrates some cortical regions-based diagnosis results of different normal as well as mildly cognitive impaired subjects. As shown in the figure, the system can visualize the disease’s severity in the cortical regions separately. In turn, this illustration helps the experts to discover any local abnormality and its degree to consequently direct the treatment plans.

## 5. Conclusions

Among the neurodegenerative conditions, AD is considered one of the leading diseases that affect the CNS, where its main sufferers are elderly people. The principal goal of the presented work is to serve the subject-dependent (i.e., personalized) diagnosis of the MCI, the early phase of AD. This goal is achieved by demonstrating a cortical region-based CAD system that helps visualize the severity of the disease in different local brain regions. Because of the difficulty of addressing the classification task between the normal and the mildly cognitive impaired groups, our system aims to target a more promising performance than in the literature and some state-of-the-art methods. To achieve this purpose, the sMRI has been used where several shape-based features were extracted, and according to the obtained results, could provide powerful assistance in the targeted task. Comparing our system with some state-of-the-art methods and validating it with the related work shows promising results of ours in the studied research area. Therefore, the proposed system can be treated as an assistant tool that provides a highly performed diagnosis through focusing on the crucial related brain regions, cortical regions. Focusing on such areas is vital due to the variable effect of AD in its sufferers that in turn requires presenting different medical services to the sufferers according to the nature of the disease’s influence and the degree of this influence in their cortical regions. Besides that, the proposed system can help analyze the disease and uncover the ambiguity surrounding it by providing a finely detailed computer-aided diagnosis system that targets the hardly discriminative early stage of the disease.

For future work, the authors plan to perform further evaluation of the presented diagnostic system with other datasets, improve the system’s overall performance, and perform additional analysis processes involving multimodal imaging to enhance the goals in this research area. Additionally, the obtained promising results that in turn helped in proofing the targeted concept of this paper, encourages using the proposed system in addressing another AD-based discrimination task that is between the sMCI and pMCI groups, and evaluating the resulting diagnosis performance for further improvements. Additionally, regarding the surface reconstruction, the authors will try to implement some other reconstruction methods and compare their results with the MC algorithm.

## Figures and Tables

**Table 1:** Demographic data relating to baseline sMRI scans selected from ADNI. Note: MMSE is the Mini Mental State Examination, and CDR is the Clinical Dementia Rating.

	60 Normal Subject	86 MCI
**Age (Mean ± std)**	75.49 ± 4.78	73.98 ± 7.72
**Gender**		
Women	38	33
Men	22	54
**MMSE scores**	24–30	24–30
**CDR**	0	0.5

**Table 2:** The person correlation for MRI parameters and distinct behavioral tasks in MCI subjects, where: BNTTOTAL: Total number correct on Boston Naming Test, BNTSPONT: number of spontaneously given correct responses, Partial Score of BNT, TOTAL11 (ADAS): total score on the 11 item cognitive subscale of the Alzheimer’s Disease Assessment Scale (ADAS), FAQTOTAL: functional assessment questionnaire total score, CONMCXLA: number of targets hit on ADNI numbers cancellation task.

Brain Region	Behavioral Task	ADNI Category	r-Value	*p*-Value
Right Angular Gyrus	Language	BNTTOTAL	0.37	0.001
Right Angular Gyrus	Language	BNTSPONT	0.36	0.001
Left Angular Gyrus	Language	BNTTOTAL	−0.35	0.002
Left Angular Gyrus	Language	BNTSPONT	−0.37	0.001
Right Middle Cingulum	Language	BNTTOTAL	−0.29	0.010
Right Middle Cingulum	Language	BNTSPONT	−0.31	0.006
Right Inferior Frontal Opercularis	Cognitive	TOTAL11 (ADAS)	−0.32	0.004
Left Parahippocampal Gyrus	Adaptive	FAQTOTAL	−0.30	0.007
Left Parahippocampal Gyrus	Visual Spatial	CONMCXLA	0.30	0.008

## Data Availability

The used data in this study was obtained from Alzheimer’s Disease Neuroimaging Initiative (ADNI). More information regarding ADNI can be obtained from the following link: http://adni.loni.usc.edu/ (accessed on 1 July 2021).

## Abbreviations

The following abbreviations are used in this manuscript:

AD	Alzheimer’s disease
CNS	central nervous system
APP	amyloid-β precursor protein
BACE1	β secretase 1
PSEN1	presenilin 1
PSEN2	presenilin 2
APH1	anterior pharynxdefective 1
MCI	mild cognitive impairment
PET	positron emission tomography
CSF	cerebrospinal fluid
A$\beta_{42}$	amyloid beta
sMRI	structural magnetic resonance imaging
FDG-PET	2-[18F] fluoro-2-deoxy-d-glucose
NC	normal control
CAD	computer-assisted diagnostic
ICA	independent component analysis
SVM	support vector machines
GA	genetic algorithms
sMCI	stable MCI
pMCI	progressive MCI
kSVM-DT	kernel support vector machine decision tree
MIL	multiple instance learning
OPLS	orthogonal partial least squares to latent structures
RDoC	research domain criteria
ADNI	Alzheimer’s disease neuroimaging initiative
MMSE	mini mental state examination
CDR	clinical dementia rating
MC	marching cubes
AAL	automated anatomical labeling
CCA	canonical correlation analysis
MNI	Montreal Neurological Institute
AC-PC	anterior and posterior commissures
pSVM	probabilistic support vector machines
BNTTOTAL	total number correct on Boston Naming Test
BNTSPONT	number of spontaneously given correct responses
ADAS	Alzheimer’s Disease Assessment Scale-Cognitive Behavior
FAQTOTAL	functional assessment questionnaire total score
CONMCXLA	number of targets hit on ADNI numbers cancellation task
RBF	radial basis function
KNN	K nearest neighbors
PCA	principal component analysis
